# Proteome profiling of *Pseudomonas aeruginosa* PAO1 identifies novel responders to copper stress

**DOI:** 10.1186/s12866-019-1441-7

**Published:** 2019-04-01

**Authors:** Bradley W. Wright, Karthik S. Kamath, Christoph Krisp, Mark P. Molloy

**Affiliations:** 1 Department of Molecular Sciences, Macquarie University, Sydney, 2109 Australia; 2 Australian Proteome Analysis Facility, Macquarie University, Sydney, 2109 Australia; 3 Present address: Bowel Cancer and Biomarker Laboratory, Kolling Instiute, The University of Sydney, Royal North Shore Hospital, Sydney, Australia

**Keywords:** Mass spectrometry, Copper stress, Proteome, *Pseudomonas aeruginosa*

## Abstract

**Background:**

The opportunistic pathogen, *Pseudomonas aeruginosa* is well known for its environmental and metabolic versatility, yet many of the functions of its gene-products remain to be fully elucidated. This study’s objective was to illuminate the potential functions of under-described gene-products during the medically relevant copper-stress condition.

**Results:**

We used data-independent acquisition mass spectrometry to quantitate protein expression changes associated with copper stress in *P. aeruginosa* PAO1. Approximately 2000 non-redundant proteins were quantified, with 78 proteins altering in abundance by +/− 1.5-fold or more when cultured to mid-log growth in the presence of 50 μM copper sulfate. One-third of those differentially expressed proteins have no prior established functional roles.

**Conclusions:**

This study provides evidence for the functional involvement of some specific proteins in enabling *P. aeruginosa* to survive under sub-lethal concentrations of copper. This further paves the way for targeted investigations into the specific mechanisms of their activity.

**Electronic supplementary material:**

The online version of this article (10.1186/s12866-019-1441-7) contains supplementary material, which is available to authorized users.

## Background

Copper is an essential trace element for all organisms, with bacteria requiring it as a co-factor to many enzymes [1, 2]. However, at high concentrations of copper it becomes toxic to the organism, the toxicity of which has been researched and exploited as an anti-microbial agent over the years [3–6]. More explicitly, there have been many studies seemingly producing strong evidence for the efficacy of incorporation of copper alloys or complexes as antimicrobial surfaces within healthcare environments [7–17]. For example, Schmidt et al. [18] studied the effect of copper-alloy commonly touched surfaces within intensive care units on microbial burden in three hospitals over 43 months. They found and concluded that the copper-alloy objects caused an 83% reduction in the average microbial burden (*p*-value < 0.0001) with respect to the control, and copper surfaces grant a safer hospital environment. A study in 2017 by Souli et al. [19] similarly studied the effects of copper-alloy coatings within the hospital environment, but focused their interests on its effects on multi-drug resistant bacteria, populations of which are prevalent in hospital environments. The sampling and analyses in this study allowed the evaluation of only clinically relevant bacteria, and found that copper-coated surfaces had a significant effect on reducing the microbial burden of multi-drug resistant gram-negative bacteria and *enterococci*. Nonetheless, many questions do remain from such studies (and importantly, future studies) such as type of copper coating, the lack of standardization, and randomization, that do need to be de-lineated before resounding conclusions can be drawn into their effectiveness (for a recent peer-reviewed commentary on this see Weber et al. [20]).

In examining the literature pertaining to the biological effects of copper exposure on bacterial species, there is a paucity of information relating to its proteome effects for organisms of clinical relevance. One of the most notable studies in the area of biological response to copper is the transcriptional profiling study of Teitzel et al. [21] on *Pseudomonas aeruginosa.* This study examined the transcriptome of copper-stressed and copper-adapted PAO1 cultures, and identified 405 and 331 differentially regulated genes (3-fold) compared to the control. Particularly, genes involved in oxidative stress response were observed in the copper stressed expression profiles, whereas genes involved in passive transport functions were observed in copper adapted cultures. Both had up-regulation of genes involved in active transport functions such as that of the Cu^2+^ transporting P-type ATPase, *yvgX* (PA3920) [21–25].

The opportunistic pathogen *P. aeruginosa* is a bacterium of high clinical significance owing to the widespread observance of infection in those that are immunocompromised. In hospital-acquired pneumonia, *P. aeruginosa* was the etiological agent in 20% of the infections [26], and is responsible for approximately 10% of all nosocomial infections [27, 28]. Of high clinical relevance is this organism’s ability to establish itself within the lungs of those with cystic fibrosis (CF). *P. aeruginosa* respiratory infection in CF patients is a chronic complication reflective of the organism’s biofilm forming [29, 30], genetic and phenotypic diversity [31–33], and multi-drug resistant capacities [34–36], as well as CF patient’s own lung pathophysiology [37, 38], all of which result in the reduced ability to rid or target the infection. Despite CF mortality rates in developed nations being at an all-time low, median age of survival is still not greater than 50 [39, 40], reflecting the continual need to explore and develop treatments.

In response to the increasing need to understand *P. aeruginosa* environmental and infectious versatility, it was one of the earliest microbes to undergo complete genome sequencing using the archetypal strain PAO1 [41]. Its large 6.3 million base pairs (Mbp) genome containing 5570 open reading frames was described as having the genetic complexity necessary to permit its wide metabolic and environmental adaptability.

In spite of the medical importance of more fully understanding the metabolic capability of *P. aeruginosa,* the functional roles of a significance number of its proteins remain poorly understood or even detected. In this study, we used modern mass spectrometry to survey the membrane and whole cell proteomes of copper-stressed *P. aeruginosa* PAO1 to discover novel copper-responsive genes. This is relevant given the heightened interest of copper compounds as bacteriostatic and bactericidal agents in clinical settings. We observed approximately 2000 proteins, positioning this as one of the deepest proteome profiling studies of this strain (see Additional file 1) and discovered 78 to be responsive to exposure to sub-lethal copper sulfate. Over 90% of these proteins have not been previously known to have a functional role in combatting copper stress.

## Results

### Current status of the PAO1 proteome

The *Pseudomonas* genome database (PGD) (http://www.pseudomonas.com) was used to review proteins identified by mass spectrometry and assign them into one of four product name confidence classes [42]. The four PGD confidence classes are: Class 1 – Function experimentally demonstrated in *P. aeruginosa*, Class 2 – Function of a highly similar gene experimentally demonstrated in another Class 3 – Function proposed based on presence of conserved amino acid motif, structural feature or limited sequence similarity to an experimentally demonstrated gene, Class 4 – Homologs of previously reported genes of unknown function, or no similarity to any previously reported genes. A review of the current PDG shows that close to 60% of PAO1 gene-products have poorly described functional roles (i.e. Class 3 and 4 confidences) (Additional file 2).

### *P. aeruginosa* protein detection using SWATH-MS

To identify hitherto unknown *P. aeruginosa* PAO1 proteins involved in response to copper stress we carried out a quantitative proteomic experiment exposing the microorganism to a sub-lethal concentration of copper sulfate (Additional file 3). Data-independent acquisition SWATH-MS [43] was used to compare both whole cell fractions and membrane preparations of control M9 media cultured microbes with those exposed to 50 μM CuSO_4._ All mass spectrometry data is reported in Additional file 4. In total, 1999 non-redundant proteins were identified (1% FDR) consisting of 1591 proteins detected from whole cell lysate fractions and 1215 from a membrane preparation. 784 and 408 proteins were uniquely identified in the whole cell fractions and membrane fractions respectively. Surprisingly, only ~ 24% of the non-redundant identified proteins mapped to the highest confidence functional assignment of PGD Class 1 proteins. Approximately 29, 15 and 31% were ascribed as Class 2, Class 3 and Class 4, respectively (Table 1). Therefore, this study has found experimental evidence for the expression of many *P. aeruginosa* PAO1 proteins which are currently annotated with very limited functional knowledge.

**Table 1:** PGD Product name confidence groupings of mass spectrometry identified proteins. Classes are as described as per PDG [42]

Class	Proteins	%
1.0	495	24.8
2.0	585	29.3
3.0	293	14.7
4.0	625	31.3
Not assigned	1	0.1
Total	1999	

A list of 1691 *P. aeruginosa* PAO1 proteins that have been experimentally confirmed or predicted (PSORTB3.0 [44]) to be membrane localized were extracted from PGD and compared to the proteins detected here (Table 2). Current estimates suggest that approximately 20–30% of genes in most microbial genomes encode for membrane proteins [45], and from the non-redundant group of proteins detected here, 589 (29.5%) were identified as membrane proteins, consistent with the accepted estimate.

**Table 2:** Shared, unique, and membrane proteins of the whole cell and membrane fractions. 1591 and 1215 were the total number of quantifiable proteins identified in the whole cell and membrane fractions respectively. There were also 589 (29.5%) non-redundant membrane proteins identified from within the 1999 proteins identified

	Whole cell fraction	Membrane fraction
Total proteins	1591	1215
Total membrane proteins	323 (20.3%)	473 (38.9%)
Proteins uniquely identified in fraction	784 (49.3%)	408 (33.6%)
Total membrane proteins uniquely observed in fraction	117 (14.9%)	266 (65.2%)

### Copper responsive proteome

SWATH-MS quantitation detected 81 proteins whose levels changed by +/− 1.5 fold or more, including 28 from the membrane-isolated fraction (Table 3) and 53 proteins in whole cell lysates upon growth in 50 μM CuSO_4_ (Table 4) – including three shared proteins.

**Table 3:** Proteins associated with copper response in *P. aeruginosa* PAO1 membrane fraction. Protein samples from the copper stressed PAO1 were compared against the control group (+/− 1.5 –fold abundance change, *p* < 0.01)

Protein	Fold change (+/−)	*p*-value	Peptides	Product Description	Subcellular Localization (localization [confidence code])*	Product Name Confidence
PA2542	42.6	0.003	1	Conserved hypothetical protein	Outer Membrane [Class 3]	Class 4
**PA3351**	20.7	0.001	1	FlgM	Extracellular [Class 3]	Class 1
PA2505	17.9	0.000	13	Tyrosine porin OpdT	Outer Membrane [Class 2]; Outer Membrane [Class 3]; Outer Membrane Vesicle [Class 1]	Class 1
PA2064	7.2	0.003	9	Copper resistance protein B precursor	Outer Membrane [Class 3]; Outer Membrane Vesicle [Class 1]	Class 2
PA3920	6.2	0.001	12	Probable metal transporting P-type ATPase	Cytoplasmic Membrane [Class 3]	Class 3
PA4492	4.7	0.006	1	MagA	Unknown [Class 3]; Cytoplasmic Membrane [Class 1]	Class 1
**PA2520**	4.3	0.007	1	Resistance-Nodulation-Cell Division (RND) divalent metal cation efflux transporter CzcA	Cytoplasmic Membrane [Class 2]; Cytoplasmic Membrane [Class 3]	Class 2
**PA5021**	2.8	< 0.0001	1	Probable sodium/hydrogen antiporter	Cytoplasmic Membrane [Class 3]	Class 3
PA4935	2.09	0.009	2	30S ribosomal protein S6	Cytoplasmic [Class 3]	Class 2
PA3730	1.67	0.009	1	Hypothetical protein	Cytoplasmic Membrane [Class 3]	Class 4
PA1766	1.64	0.006	4	Hypothetical protein	Cytoplasmic [Class 3]	Class 4
PA3459	−1.50	0.005	4	Probable glutamine amidotransferase	Cytoplasmic [Class 3]	Class 3
PA3731	−1.53	0.005	6	Conserved hypothetical protein	Cytoplasmic [Class 3]	Class 4
PA5174	−1.56	0.007	4	Probable beta-ketoacyl synthase	Cytoplasmic [Class 3]	Class 3
PA3011	−1.58	0.009	7	DNA topoisomerase I	Cytoplasmic [Class 3]	Class 2
PA5204	−1.67	0.002	12	N-acetylglutamate synthase	Cytoplasmic [Class 3]	Class 1
PA3552	−1.79	0.007	5	ArnB	Cytoplasmic [Class 3]	Class 2
PA4272	−1.86	0.001	3	50S ribosomal protein L10	Cytoplasmic [Class 3]	Class 2
PA3769	−1.95	0.001	6	GMP synthase	Cytoplasmic [Class 3]	Class 2
PA0390	−2.05	0.010	2	homoserine O-acetyltransferase	Cytoplasmic [Class 3]	Class 2
PA3042	−2.14	0.001	3	Hypothetical protein	Unknown [Class 3]	Class 4
PA5312	−2.14	0.008	7	Aldehyde dehydrogenase	Cytoplasmic [Class 3]	Class 2
PA3040	−2.21	0.002	4	Conserved hypothetical protein	Unknown [Class 3]; Outer Membrane Vesicle [Class 1]	Class 4
PA2345	−2.35	0.002	6	Conserved hypothetical protein	Unknown (This protein may have multiple localization sites) [Class 3]; Unknown (This protein may have multiple localization sites) [Class 3]	Class 4
**PA3952**	−2.35	0.002	2	Hypothetical protein	Unknown [Class 3]	Class 4
PA3746	−2.70	0.001	2	Signal recognition particle protein Ffh	Cytoplasmic Membrane [Class 3]	Class 2
PA4786	−3.15	0.002	6	Probable short-chain dehydrogenase	Cytoplasmic [Class 2]; Cytoplasmic [Class 3]	Class 3
PA3183	−3.50	0.008	4	Glucose-6-phosphate 1-dehydrogenase	Cytoplasmic [Class 3]	Class 1

*Extracted from PGD. Class 1: Subcellular localization experimentally demonstrated in *Pseudomonas aeruginosa*. Class 2: Subcellular localization of a similar gene experimentally demonstrated in another organism OR to a paralog experimentally demonstrated in the same organism. BLAST expect value of 10e-10 for query within 80–120% of subject length. Class 3: Subcellular localization computationally predicted by PSORTb [44]. **Bold** indicates first time evidence of protein translation in PAO1

**Table 4:** Proteins associated with copper response in *P. aeruginosa* PAO1 whole cell lysate. Protein samples from the copper stressed PAO1 were compared against the control group (+/− 1.5 –fold abundance change, *p* < 0.01)

Protein	Fold change (+/−)	*p*-value	Peptides	Product Description	Subcellular Localization (localization [confidence code])*	Product Name Confidence
**PA3661**	63.0	< 0.0001	2	Hypothetical protein	Unknown [Class 3]	Class 4
PA2505	25.1	< 0.0001	10	Tyrosine porin OpdT	Outer Membrane [Class 2]; Outer Membrane [Class 3]; Outer Membrane Vesicle [Class 1]	Class 1
**PA4714**	16.2	0.002	5	Conserved hypothetical protein	Unknown (This protein may have multiple localization sites) [Class 3]; Unknown (This protein may have multiple localization sites) [Class 3]	Class 4
PA2064	10.8	< 0.0001	6	Copper resistance protein B precursor	Outer Membrane [Class 3]; Outer Membrane Vesicle [Class 1]	Class 2
**PA2405**	10.3	0.003	1	FpvJ	Unknown [Class 3]	Class 1
**PA3412**	10.2	0.005	3	Hypothetical protein	Unknown [Class 3]	Class 4
**PA2807**	10.1	0.004	4	Hypothetical protein	Unknown [Class 3]	Class 4
**PA4224**	6.6	0.002	6	Pyochelin biosynthetic protein PchG	Cytoplasmic [Class 3]	Class 1
**PA3445**	6.2	0.002	3	Conserved hypothetical protein	Unknown (This protein may have multiple localization sites) [Class 3]; Unknown (This protein may have multiple localization sites) [Class 3]	Class 4
**PA4884**	4.9	0.004	4	Hypothetical protein	Periplasmic [Class 3]	Class 4
PA2065	4.4	< 0.0001	8	Copper resistance protein A precursor	Periplasmic [Class 2]; Periplasmic [Class 3]; Outer Membrane Vesicle [Class 1]	Class 2
PA3920	4.0	0.003	2	Probable metal transporting P-type ATPase	Cytoplasmic Membrane [Class 3]	Class 3
PA3182	3.0	0.008	2	6-phosphogluconolactonase	Unknown [Class 3]	Class 1
PA0283	2.8	0.009	10	Sulfate-binding protein precursor	Periplasmic [Class 2]; Periplasmic [Class 3]	Class 2
PA3450	2.8	0.001	6	Probable antioxidant protein	Cytoplasmic [Class 3]	Class 3
**PA0346**	2.5	0.004	6	Hypothetical protein	Unknown [Class 3]	Class 4
PA4923	2.3	0.010	2	Conserved hypothetical protein	Unknown [Class 3]	Class 4
PA3165	2.0	0.007	5	Histidinol-phosphate aminotransferase	Cytoplasmic [Class 3]	Class 2
PA5215	1.9	0.006	1	Glycine-cleavage system protein T1	Cytoplasmic [Class 3]	Class 2
PA5335	1.7	0.005	1	Conserved hypothetical protein	Cytoplasmic [Class 3]	Class 4
PA4240	1.7	0.009	3	30S ribosomal protein S11	Cytoplasmic [Class 3]; Outer Membrane Vesicle [Class 1]	Class 2
PA0473	1.7	0.006	2	Probable glutathione S-transferase	Cytoplasmic [Class 3]	Class 3
PA5188	1.7	0.010	2	Probable 3-hydroxyacyl-CoA dehydrogenase	Cytoplasmic [Class 3]	Class 3
PA3029	1.6	0.007	5	Molybdopterin biosynthetic protein B2	Cytoplasmic [Class 3]	Class 2
PA4110	1.6	0.010	5	Beta-lactamase precursor (AmpC)	Periplasmic [Class 1]; Periplasmic [Class 3]	Class 1
**PA1024**	1.6	< 0.0001	1	2-Nitropropane Dioxygenase	Cytoplasmic [Class 3]	Class 1
PA3896	1.6	0.001	2	Probable 2-hydroxyacid dehydrogenase	Cytoplasmic [Class 2]; Cytoplasmic [Class 3]	Class 3
PA4947	1.5	0.007	1	N-acetylmuramoyl-L-alanine amidase	Unknown [Class 3]	Class 2
PA1151	1.5	0.005	3	Pyocin S2 immunity protein	Cytoplasmic [Class 3]	Class 1
PA3243	1.5	0.001	3	Cell division inhibitor MinC	Cytoplasmic [Class 3]	Class 2
PA2572	−1.6	0.008	1	Probable two-component response regulator	Cytoplasmic [Class 3]	Class 3
PA0833	−1.6	0.008	2	Hypothetical protein	Outer Membrane [Class 3]; Outer Membrane Vesicle [Class 1]	Class 4
PA2476	−1.7	0.008	3	Thiol:disulfide interchange protein DsbG	Periplasmic [Class 3]	Class 2
PA5209	−1.9	0.001	2	Hypothetical protein	Unknown (This protein may have multiple localization sites) [Class 3]; Unknown (This protein may have multiple localization sites) [Class 3]	Class 4
PA5562	−1.9	0.002	5	Chromosome partitioning protein Spo0J	Cytoplasmic [Class 3]	Class 2
PA0265	−2.0	0.004	3	Succinate-semialdehyde dehydrogenase	Cytoplasmic [Class 3]	Class 2
PA5554	−2.0	< 0.0001	15	ATP synthase beta chain	Cytoplasmic [Class 3]; Outer Membrane Vesicle [Class 1]	Class 2
PA2259	−2.1	0.006	1	Transcriptional regulator PtxS	Cytoplasmic [Class 3]	Class 1
PA4317	−2.2	0.006	1	Hypothetical protein	Cytoplasmic Membrane [Class 3]	Class 4
PA4764	−2.3	0.008	2	Ferric uptake regulation protein	Cytoplasmic [Class 1]; Cytoplasmic [Class 3]	Class 1
PA1580	−2.3	0.009	5	Citrate synthase	Cytoplasmic [Class 3]	Class 1
PA4360	−2.4	0.008	2	Hypothetical protein	Unknown [Class 3]; Outer Membrane Vesicle [Class 1]	Class 4
PA1608	−2.5	0.008	2	Probable chemotaxis transducer	Cytoplasmic Membrane [Class 3]; Outer Membrane [Class 2]	Class 3
PA3214	−2.7	0.002	2	Hypothetical protein	Unknown [Class 3]	Class 4
PA0762	−2.7	0.007	2	Sigma factor AlgU	Cytoplasmic [Class 3]	Class 1
PA0122	−2.8	0.003	4	rahU	Extracellular [Class 3]	Class 1
PA4310	−2.9	0.004	3	Chemotactic transducer PctB	Cytoplasmic Membrane [Class 3]	Class 1
PA2654	−3.2	0.008	6	Probable chemotaxis transducer	Cytoplasmic Membrane [Class 3]	Class 3
PA2815	−4.2	0.006	4	Probable acyl-CoA dehydrogenase	Cytoplasmic Membrane [Class 3]; Outer Membrane Vesicle [Class 1]	Class 3
PA4876	−4.7	0.003	2	Osmotically inducible lipoprotein OsmE	Unknown [Class 3]; Outer Membrane Vesicle [Class 1]	Class 2
PA1324	−6.1	0.003	5	Hypothetical protein	Unknown [Class 3]	Class 4
PA3691	−6.2	0.009	8	Hypothetical protein	Unknown [Class 3]; Outer Membrane Vesicle [Class 1]	Class 4
PA3110	−16.7	< 0.0001	1	Hypothetical protein	Unknown [Class 3]	Class 4

*Extracted from PGD. Class 1: Subcellular localization experimentally demonstrated in *Pseudomonas aeruginosa*. Class 2: Subcellular localization of a similar gene experimentally demonstrated in another organism OR to a paralog experimentally demonstrated in the same organism. BLAST expect value of 10e-10 for query within 80–120% of subject length. Class 3: Subcellular localization computationally predicted by PSORTb [44]. **Bold** indicates first time evidence of protein translation in PAO1

**Fig. 1:**
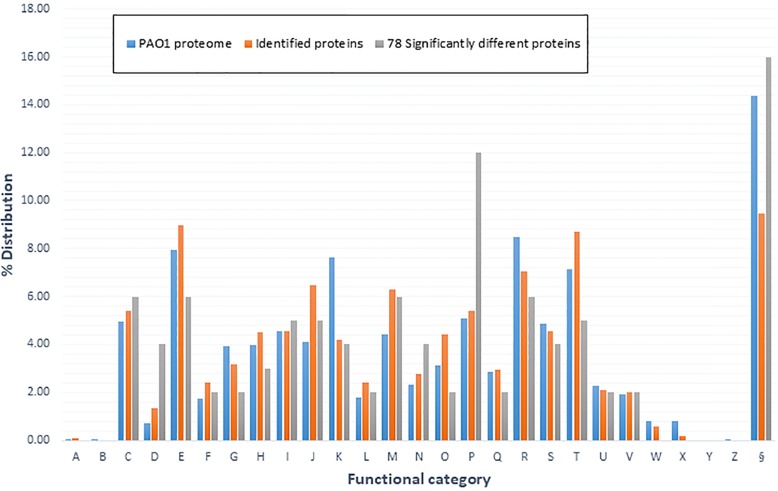
COG functional categories used to represent major biological functions. This distribution map highlights the COG % distribution for all non-redundant proteins from this experiment and 78 proteins deemed significantly differentially regulated. This also contains the % distribution for 5698 proteins of PAO1 – PAO1 proteome (derived from the PGD). The COG functional categories are: **a**– RNA processing and modification, **b**– chromatin structure and dynamics, **c**– energy production and dynamics, **d**– cell cycle control, cell division, and chromatin partitioning, **e**– amino acid transport and metabolism, **f**– nucleotide transport and metabolism, **g**– carbohydrate transport and metabolism, **h**– coenzyme transport and metabolism, **i**– lipid transport and metabolism, **j**– translation, ribosomal structure, and biogenesis, **k**– transcription, **l**– replication, repair, and recombination, **m**– cell wall/membrane/envelope biogenesis, **n**– cell motility, **o**– post-translational modification, protein turnover, and chaperones, **p**– inorganic ion transport, **q**– secondary metabolite biosynthesis, transport, and catabolism, **r**– general function prediction only, **s**– function unknown, **t**– signal transduction mechanisms, **u**– intracellular trafficking, secretion, and vesicular transport, **v**– defense mechanisms, **w**– extracellular structures, **x**– mobilome: prophages, and transposons, **y**– nuclear structure, **z**– cytoskeleton, and **§** – no COG assignment

To investigate putative biological functions of the copper-response proteins we conducted bioinformatics analysis placing genes into 26 possible clusters of orthologs (COGs) biological functions (Fig. 1, Additional file 5) [46].

As seen in Fig. 1 almost all categories had some level of proteins that were differentially regulated by copper exposure, but some more than others. Unsurprisingly, COG (P) the category for inorganic ion transport contained the highest number of copper-regulated proteins, compared with M9 control cells. Categories E (amino acid transport and metabolism), F (nucleotide transport and metabolism), G (carbohydrate transport and metabolism), H (coenzyme transport and metabolism), Q (secondary metabolite biosynthesis, transport and catabolism), and O (post-translational modification, protein turnover, and chaperones) were observed to have lower normalised distributions than that of the PAO1 proteome. Conversely, D (cell cycle control, cell division, and chromatin partitioning), and N (cell motility) are observed to have higher normalised distributions than that of PAO1 proteome. Motility is an essential adaptive response and a number of chemotaxis or probable chemotaxis related proteins were observed to be significantly down-regulated (Table 4). Others have previously observed a similar trend of chemotaxis inhibition in *Escherichia coli* when cultured in the presence of Cu^2+^ [47, 48]. We were surprised to observe a lower normalized distribution of proteins in the COG related to signal transduction mechanisms (T) as it is well reported in literature that cells utilize various signal transduction pathways in response to metal-induced oxidative stress [49–53].

## Discussion

To further understand the biological response of *P. aeruginosa* to copper exposure we quantitatively examined the proteome using advanced mass spectrometry. This enabled the identification of approximately, 2000 proteins, including several new copper-responsive proteins not previously associated with copper stress and highlighting them for future functional analyses (Tables 3 and 4).

### Novel copper responsive proteins

We observed 78 proteins whose abundances significantly altered upon copper exposure including three known copper-responsive proteins (Cu^2+^ transporting P-type ATPase, PA3920, and the copper binding and sequestering proteins PA2064 (CopB) and PA2065 (CopA) [54–57]), confirming that the microorganism was under copper-mediated stress. In addition, seven of the 78 differentially regulated proteins were observed to be transcriptionally regulated in the copper-response transcriptome study of Teitzel et al [21] (Additional file 6). Thus, for these proteins, there is compelling evidence to support their role as copper-responsive proteins.

Amongst the novel findings, PA2807 is a 22.5 kDa, PGD confidence Class 4 protein which has not been described in other functional experiments. According to the InterPro database, this protein contains two domains: an EfeO-type cupredoxin-like domain and a Blue (type 1) copper domain. *PA2807* gene is located adjacent to *PA2808*, a gene observed to be upregulated in response to copper and is described as a *Pseudomonas* type III repressor [58, 59]. Furthermore, a two-component copper regulatory system dubbed *CopR/S* (*PA2809*, and *PA2810)* lies immediately upstream of *PA2808* and *PA2807*. PA2809 and PA2810 have previously been observed to be up regulated in the presence of copper [21, 60, 61], and when *PA2809* is knocked-out, the organism exhibits higher sensitivity to copper [59, 61]. In our study, PA2807 was highly induced (10-fold) while PA2809 and PA2810 did not satisfy statistical significance, and PA2808 was not observed. Nonetheless, the combined evidence strongly supports a role for PA2807 in the copper response system in *P. aeruginosa* as a member of the plastocyanin/azurin copper-binding family [58], and can be reclassified in PGD.

Under copper-mediated stress, there were a considerable number of proteins observed to be differentially regulated that are currently not well annotated, nor have sufficient functional predictions available to confidently assign a biological function. Of particular note was the protein PA2542 which showed a > 40-fold level of induction upon copper stress. This large 130.5 kDa predicted outer membrane protein, holds some homology at its C-terminus (amino acids 900–1221) to the *E. coli* TamB family that forms a complex with TamA, resulting in the TAM complex, a recently described novel protein secretion system [62]. If PA2542 is in fact a homolog of TamB our study provides novel evidence for the reliance of *P. aeruginosa* for utilising the TAM transport system in responding to copper exposure. The TAM system’s crucial function in assembly of outer membrane proteins [63, 64] (and our observation of the strong up-regulation of PA2542) directly aligns with the high fold changes shown in Tables 3 and 4 of proteins whose annotated subcellular localisation is that of the outer membrane. It is of no surprise these include copper-stress related membrane proteins PA3920, PA2064, and PA2065 (among others).

In Table 4, we observed large fold change inductions for a number of other proteins classed as hypothetical proteins, including, PA3661 (+ 63.0), PA4714 (+ 16.2), and PA3110 (− 16.7). PA3661 is a predicted small, 12.3 kDa protein that has no known functional role, but is likely to be localised to the outer membrane due to prediction of a type II non-cytoplasmic signal peptide [44, 65]. The fact that it is induced so strongly as a result of copper exposure paints a role in Cu^2+^ resistance and makes this an interesting lead to pursue. Other lipoproteins, PA1324, PA3691, and PA4876, were observed to be down-regulated (Table 4), consistent with the decreased expression of their regulator, sigma factor AlgU (also known as AlgT and σ^22^) (Table 4) [66, 67].

Hypothetical protein PA4714 shares homology with a poorly defined family of proteins collectively named DUF411, a group in which there is little insight into function other than possible cation resistance and/or metal binding activity. This proposed activity aligns with the response we observed to copper-mediated stress. The conserved hypothetical protein PA3110, was observed to be significantly down regulated in response to copper stress. Investigating further, InterPro, and EggNOG protein analysis [68], confidently highlighted a sporulation-like domain (SPOR) on the C-terminus of PA3110. SPOR domains are found in thousands of bacterial proteins [69], and work performed studying SPOR domain containing proteins in *E. coli* have found that these domains are involved in binding to peptidoglycan at the septal ring [70–72], and thus SPOR domains provide targeting mechanisms for their respective proteins.

### Function refinement

Upon copper exposure we observed up regulation of the iron homeostasis protein, PchG (PA4224, Table 4). This protein is reliably characterised as a key protein in the synthesis of the iron siderophore pyochelin [73] and is produced and secreted by *P. aeruginosa* PAO1 to chelate Fe^3+^ and transport it back to the cell. PchG has also been shown to chelate other metals, including Cu^2+^, albeit with lower affinities [74, 75]. In further agreement to our observation we detected the down regulation of PA4764 (Ferric uptake regulator - Fur), a protein whose expression negatively controls the production of pyochelin by indirectly acting on its biosynthetic genes [76]. Together, our data support a role for PchG in binding Cu^2+^. We further noted the changed expression of other proteins involved in pyochelin activity from the whole cell fraction (Additional file 1). PA4221 (+ 4.1 fold, *p*-value 0.01), PA4225 (+ 3.1 fold, *p*-value 0.02), PA4226 (+ 4.2 fold, *p*-value 0.06), PA4227 (+ 1.6, *p*-value 0.33), PA4228 (+ 3.4 fold, *p*-value 0.02), and PA4229 (+ 3.3 fold, *p*-value 0.03), although not all passed our statistical cut-off threshold.

On first thought the observations of Teitzel et al. [21], and Visca et al. [75] seem to fit with assumption that the siderophore pyochelin would be suppressed under copper exposure due to its inherent ability to chelate it and presumably then transport it into the cell. However, to somewhat rationalise and explain our contrary results we highlight the study of Braud et al. [74], in which they found that pyochelin chelated to 16 metals (including Cu^2+^) and all but Hg^2+^ bound to the specific outer membrane transporter FptA (PA4221), a transporter that facilitates the transfer of ferric iron into the cell. Yet, FptA metal uptake into the cell was only specific for Fe^3+^, Co^2+^, Ga^3+^, and Ni^2+^ [74], and that in the absence of pyochelin in the extracellular medium, sensitivity to Cu^2+^ was increased. There is a clear need for further study to clarify the difference we observed, however, it is clear that pyochelin provides a reduction in Cu^2+^ toxicity through its chelation and decreased diffusion through the cell [74] and this may be described as a beneficial pseudo-function.

PA2505, OpdT was observed to be strongly up regulated in the whole cell and membrane fractions and is described as a tyrosine porin. In the study by Tamber et al. [77], they suggest that some sub-members of the OprD family of porins, which includes OpdT, are likely candidates for permitting low levels of non-specific uptake through the outer membrane. Therefore, it is quite possible that this porin encoding gene may be involved in secretion of small molecules and ions across the membrane, and thus may have an integral role in copper stress response through passive diffusion. OpdT has also been observed to be upregulated in copper adapted cells [21], where it was suggested that the induction of *OpdT* compensates for the repression of the other sub-family members of the OprD family while not compromising the cell.

### Virulence and pathogenesis

The membrane transporter, PA2520 (CzcA) is a heavy metal cation efflux protein which we observed in the membrane proteomic data to be induced 4.3-fold when exposed to copper. Previous studies have strongly implicated the upregulation of CzcCBA in *P. aeruginosa* with carbapenam resistance [60, 78, 79]. This effect stems from the decreased expression of the OprD porin (which we did not observe) that has been correlated to the overexpression of the Czc system, and therefore results in the decreased diffusion of antibiotics into the cell [79]. Similarly, OpdT which is known to be positively regulated under this same system [80] was also increased in abundance under copper-stress. The significance of these findings highlights potential concerns of efficacy of using copper-coated surfaces as bactericidal agents.

Interestingly, we noted that the proteins corresponding to the pyoverdine siderophore system were unchanged, except one protein, PA2405 (FpvJ) which is up-regulated. This protein contains a signal peptide supporting a periplasmic location and its genome organisation suggested it is probably involved in the transport of Fe2+ after reduction of iron from ferri-pyoverdine [81].

We detected additional proteins regulated by copper exposure that are involved in or predicted to be involved in virulence or pathogenesis. An example is MagA which was which has a fold change induction under copper exposure of 4.7. The *magABCDEF* operon has been described as encoding a structural homolog of the human α2-macroglobulin, a large-spectrum protease inhibitor [82], but the precise role of *MagA* is unclear. Here, we show it is induced by copper exposure and further studies will be needed to determine its exact function. Similarly to MagA, AmpC (PA4110) was also up-regulated in the presence of copper and has been described as a chromosomal encoded cephalosporinase [83, 84]. A study by Cabot et al. [83] investigating the prevalence of *ampC* overexpression in *P. aeruginosa* isolates from bloodstream infections, showed a strong relationship to β-lactam and aminoglycoside resistance with *ampC* overexpression. Worryingly, they noted that 100% of isolates resistant to all first-line β-lactams and aminoglycosides had an *ampC* overexpression phenotype [83]. It is unclear why copper-mediated stress would induce this cephalosporinase, and therefore further investigation is required.

### Conclusion

This study examined the proteome of *P. aeruginosa* PAO1, a common nosocomial microbe and biofilm-forming species in response to copper ion exposure. This facilitated the identification of 78 proteins as responders to copper stress, including a number of proteins not previously recognized for a role in copper response. It is important to understand the effect specific metals have on pathogenic bacterial species as copper alloys are of considerable interest to the medical microbiology community for their potential role as agents to inhibit the attachment and growth of bacterial species on commonly handled surfaces within hospital environments.

## Methods

### Cultivation

All equipment, media, and water (milliQ) were subjected to sterilization by autoclaving (121 °C, and 15 psi for 20 min) prior to experimental work. The *P. aeruginosa* strain used for this study was PAO1 and for cultivation it was grown on Luria Broth (LB) agar plates (NaCl 10 g/L, tryptone 10 g/L, yeast extract 2.5 g/L, and agar 15 g/L), and in M9 liquid medium (11.28 g/L 1x M9 salts (Sigma Aldrich), 0.24 g/L MgSO_4_, 0.01 g/L CaCl_2_, and 3.6 g/L). Cultures were taken from − 80 °C stock storage and depending on requirements were either streaked for isolated colonies onto LB plates and grown for 18–24 h at 37 °C, or cultured into 10 mL of M9 and grown for 14–16 h at 37 °C and 200 rpm agitation. The cultured PAO1 was used for the basis of further experimental work.

### Growth curve

The flask growth study using PAO1 was conducted in 250 mL conical flasks with 50 mL M9 cultures (1/100 dilution) in triplicate with or without 50 μM CuSO_4_ (CuSO_4_.5H_2_O ≥ 98%, Sigma Aldrich). Cultures were subjected to 37 °C and 150 rpm agitation, with optical density readings (OD_600_) taking hourly from the fourth hour of growth until the 20-first hour of growth (Additional file 3).

### Sample preparation

Sample preparation was adapted from the methods of Molloy, M.P. [85]. Growth of PAO1 was conducted in 400 mL (1/100 dilution) of M9 in 2 L flasks at 37 °C and 150 rpm. PAO1 was grown in triplicate with or without the addition of 50 μM CuSO_4_.

Each culture was harvested at mid-log growth by pelleting the medium via centrifugation at 3500 g at 4 °C for 8 mins. The supernatant was discarded and the pellet was washed with phosphate buffered saline (140 mM NaCl, 2.7 mM KCl, 10 mM phosphate buffer pH 7.4) (PBS)), followed by centrifugation at 3900 g at 4 °C for 8 minutes, and pellet collection in a 2 mL screw cap tube. Ice-cold PBS was added to the tubes (500-700 μL), as well as protease inhibitor (Roche), and benzonase (Sigma Aldrich). Three cycles of bead beating was performed to lyse the cells using acid-washed 180 μm (16–25 U.S. sieve) glass beads (Sigma Aldrich) at intensity 4.5 for 10 s with intermittent cooling on ice for 10 min between each cycle. Centrifugation of samples was then performed at 2500 g at 4 °C for eight minutes and a supernatant from each was collected and retained.

Sodium carbonate based membrane protein extraction was performed on a portion of supernatant from each of the samples. The supernatants to be used were pooled for each of the triplicates and were added to 33 mL of ice-cold 100 mM sodium carbonate and were placed on a rocker in 4 °C for one hour. The solutions then underwent ultracentrifugation at 115,000 g at 4 °C for one hour, after which the supernatant was discarded. PBS was then added to the tubes and the ultracentrifugation procedure was repeated. Reconstitution of the pellet was performed with 60 μL of 100 mM TEAB buffer with 1% sodium deoxycholate followed by three cycles of vortex and sonication in a water bath for 15 min at 4 °C, putting the tubes on ice intermittently. The solution was then placed into 2 mL protein low-bind tubes.

In-solution digestion of proteins was performed for membrane and soluble fractions. Reduction and alkylation of cysteine residues was carried out by incubation with 10 mM of dithiothreitol at 60 °C for 30 min, followed by incubation with 20 mM of iodoacetamide at room temperature, in the dark for 20 min. Trypsin was added at a 1:50 ratio of trypsin to protein (*w*/w) and left to incubate at 37 °C overnight. 100% formic acid is then added to the samples at a final concentration of 2% formic acid to precipitate out the sodium deoxycholate, that is then subsequently removed, and finally the samples are freeze-dried at − 20 °C for mass spectrometry.

### Mass spectrometry

All mass spectrometry runs were performed using a TripleTOF 6600 mass spectrometer with an ekspert™ nanoLC400 system with cHipLC system (SCIEX). Peptide retention occurred through a reverse phase 200 μm × 0.5 mm cHiPLC® trap column (3 μm, 120 Å pore size C18-CL), and a 15 cm × 200 μm cHiPLC® analytical column (3 μm, 120 Å pore size C18-CL). Freeze-dried tryptic peptide samples were re-suspended in solvent A (2% acetonitrile, 0.1% formic acid). Re-suspension in solvent A allows the hydrophobic sections of the peptides to bind to a reverse-phase column. 3 μg (10 μL) of sample was injected onto the trap using an auto-sampler, and peptide elution from the reverse-phase column was achieved with solution A, and solution B (95% acetonitrile, 0.1% formic acid) 600 μL/min elution gradient over a 140-min chromatographic runtime. The elution gradient was as follows: from 5% solvent B to 40% solvent B within 120 min, from 40% B to 85% B within 2 min, 85% for 5 min and 5% B for 10 min. Electrospray ionization was used at a spray voltage of 2500 V to produce positively charged ions for MS/MS.

### IDA-MS

Spectra were collected for the 20 most intense precursor ions (with charge state + 2 to + 4) across the *m/z* range of 350–1500 (accumulation time 250 ms). To reduce redundant precursor selection, a dynamic exclusion of 30 s was used. MS/MS spectra were collected from 100 to 1800 *m/z* with 100 ms accumulation time.

Spectra were searched against a *P. aeruginosa* whole protein dataset derived from the PGD (12–08-14) using the ProteinPilot™ software 5.0 (SCIEX). Proteins and peptides were accepted with a protein false discovery rate of < 1%. Carbamidomethylation of cysteine residues was set as a fixed modification.

### SWATH-MS

All precursor ions were selected for fragmentation within each of 100 variable windows (Additional file 7) a *m/z* range of 400–1250 within a total cycle time of 3.1 s. Collision energies were calculated for a doubly (+ 2) charged species with a *m/z* of lowest mass in window + 10% window size. MS/MS spectra were collected across the *m/z* range of 350–1500 with a 35 ms accumulation time.

### Data analysis

SWATH-MS data was extracted using Peakview v2.1 with SWATH Micro-App v2.0 (SCIEX). A spectral library generated from the membrane, and soluble fraction IDA runs was used to extract information from the SWATH-MS runs. Data processing in Peakview v2.1 was as follows: number of peptides per protein – max. 100, number of transitions per peptide – 6, peptide confidence level – 99%, transition false discovery rate < 1%, 10-min extraction window and fragment extraction tolerance of 75 ppm.

Peakview v2.1 results were loaded into and processed in Perseus v1.5.2.6 (Max Planck Institute of Biochemistry). Peak area data (area under the curve) across all samples and proteins were normalized (division with grouping’s calculated median), and log2 transformed. Student’s t-test (two-tailed, homoscedastic) was performed in Perseus to obtain a *p*-value to determine statistical significance. Peak area was averaged between triplicates for individual proteins, and conditions (+/− CuSO_4_) and fold change of proteins were calculated between CuSO_4_ stressed and the control group and shown in Additional file 4. Additional analyses was performed in Microsoft Excel (Microsoft) using data from the *Pseudomonas* genome database (PGD) and data exported from the Perseus analysis, which included clusters of orthologous groups, product class and subcellular localization analyses.

## Additional files

Additional file 1: A comprehensive list of *P. aeruginosa* PAO1 proteomic studies of the period 2000–2017. (DOCX 71 kb)

Additional file 2: *P. aeruginosa* PAO1 gene product distributions. (DOCX 596 kb)

Additional file 3: *P. aeruginosa* growth curve under exposure to CuSO_4_. (DOCX 17 kb)

Additional file 4: Mass spectrometry data. (XLSX 2115 kb)

Additional file 5: Proteins mapped to COG functional categories that are used to represent major biological functions. (DOCX 1388 kb)

Additional file 6: Shared observations of this study (proteomic) and the transciptomic study of Teitzel et al. (DOCX 25 kb)

Additional file 7: SWATH-MS variable windows across the 400–1250 *m/z* range. (DOCX 16 kb)

## Abbreviations

COG: Cluster of orthologous group
FDR: False discovery rate
IDA: Information dependent acquisition
PDG: *Pseudomonas* genome database
SPOR: Sporulation-type domain
SWATH-MS: Sequential windowed acquisition of all theoretical mass spectra
