# Supplementary material for: Proteome profiling of Pseudomonas aeruginosa PAO1 identifies novel responders to copper stress
Source: BMC Microbiol. 2019 Apr 1;19:69. doi: 10.1186/s12866-019-1441-7 (PMC6444534; doi:10.1186/s12866-019-1441-7)
Supplement: Supplementary file 1 — A comprehensive list of P. aeruginosa PAO1 proteomic studies of the period 2000–2017. (DOCX 71 kb) [file 12866_2019_1441_MOESM1_ESM.docx]

**Additional file 1: PAO1 proteomic studies of the period 2000-2017.**

| PMID  (Reference) | Year | PAO1 Proteome  (Condition)* | Proteins identified^¶^ | Method of identification |
| --- | --- | --- | --- | --- |
| 11271498 ^(1)^ | 2000 | Whole membrane | 189 | 2-DE and MALDI-TOF MS |
| 11092861 ^(2)(2)(2)^ | 2000 | Soluble extract: PAO1 wild-type and PAO1 with *mucA22* allele | 4 | 2-DE and amino-terminal sequence analysis |
| 12362351 ^(3)^ | 2002 | Outer membrane and secretome | 32, 166 | 2-DE, MALDI-TOF MS and ESI-MS/MS |
| 12724392 ^(4)^ | 2003 | Extracellular: PAO1 wild-type and PAO1 quorum sensing mutants | 31 | 2-DE and MALDI-TOF MS |
| 14641572 ^(5)(5)(5)^ | 2003 | Soluble extract  (with and without iron-limitation) | 25^§^ | 2-DE and MALDI TOF |
| 15253424 ^(6)(6)(6)^ | 2004 | Whole membrane | 707 | LC-MS/MS |
| 15817780 ^(7)(7)(7)^ | 2005 | Extracellular  (iron-limiting and iron-rich conditions) | 26 | 2-DE and MALDI-TOF MS |
| 16641435 ^(8)(8)(8)^ | 2006 | Soluble extract  (Exposure and non-exposure to azithromycin) | 43 | 2-DE and MALDI-TOF |
| 16622056 ^(9)(9)(9)^ | 2006 | Soluble extract  (Grown with glucose or succinate, with or without glycine betaine) | 21 | 2-DE and MALDI-TOF |
| 17975093 ^(10)(10)(10)^ | 2007 | Cytoplasm  (CaCl_2_.2H_2_O – biofilm and planktonic cultures) | 146 | 2-DE and MALDI-TOF/TOF |
| 19333994 ^(11)^ | 2009 | Periplasm | 395 | 2-DE and MALDI-TOF/TOF |
| 21751344 ^(12)^ | 2011 | Outer membrane vesicles | 338 | LC-MS/MS |
| 21631603 ^(13)(13)(13)^ | 2011 | Soluble extract  (ammonium metavanadate exposure and non-exposure) | 17^§^ | 2-D LC-MALDI-TOF/TOF |
| 22950023 ^(14)^ | 2012 | Cytoplasm | 159 | 2-DE and MALDI-TOF |
| 22909304 ^(15)(15)(15)^ | 2012 | Soluble extract and biofilm matrix | 942 | SDS-PAGE and LC-MS/MS |
| 22210761 ^(16)(16)(16)^ | 2012 | Soluble extract: PAO1 wild-type, PAO1 *kinB* and PAO1 *kinB* Δ*rpoN* | 121 | MALDI-TOF/TOF |
| 22188372 ^(17)(17)(17)^ | 2012 | Soluble extract  (Pulsed light-induced stress exposure and non-exposure) | 15 | 2-DE and MALDI-TOF/TOF |
| 22264352 ^(18)(18)(18)^ | 2012 | Soluble extract | 1700 | 2-DE, MALDI-TOF MS and 2-DLC-MS/MS |
| 23744604 ^(19)^ | 2013 | Cytoplasmic membrane | 991 | SDS-PAGE and LC-MS/MS |
| 23869205 ^(20)(20)(20)^ | 2013 | Soluble extract  (ZSB adapted and ZSB non-adapted) | 8 | 2D-DIGE and LC-MS/MS |
| 23154974 ^(21)(21)(21)^ | 2013 | Soluble extract  (GABA exposure and non-exposure) | 7 | 2-DE and MALDI-TOF/TOF |
| 24291602 ^(22)(22)(22)^ | 2014 | Soluble extract: PAO1 wild-type, and PAO1 *ampR* deletion mutant (benzyl penicillin exposure and non-exposure) | 2965 | SDS-PAGE and LC-MS/MS |
| 25232353 ^(23)(23)(23)^ | 2014 | Soluble extract and outer membrane vesicle resistance sub-proteome  (biofilm and planktonic cultures at three time points) | 50^§^ | LC-MS/MS |
| 24918783 ^(24)(24)(24)^ | 2014 | Soluble extract: PAO1 *efhP* deletion mutant  (CaCl_2_.2H_2_O – biofilm and planktonic cultures) | 15^§^ | 2-DE and MALDI-TOF/TOF |
| 24742327 ^(25)(25)(25)^ | 2014 | Soluble extract | 1692 | LC-MS/MS |
| 24549289 ^(26)(26)(26)^ | 2014 | Soluble extract  (TiO_2_ EVOH particles photo-catalysis) | 1137 | LC-MS/MS |
| 24532839 ^(27)(27)(27)^ | 2014 | Soluble extract  (biofilm and planktonic cultures at three time points) | 1884 | LC-MS/MS |
| 25317949 ^(28)^ | 2015 | Whole cell - soluble and insoluble fraction: PAO1 wild-type, and PAO1 Fap overexpressing derivative | 2801 | SDS-PAGE and LC-MS/MS |
| 26303878 ^(29)^ | 2015 | Outer membrane vesicle and biofilm matrix | 466 | 2D LC-MS/MS and 2DE LC-MS/MS |
| 26431321 ^(30)^ | 2015 | Soluble extract | 1353 | SDS-PAGE and LC-MS/MS (SWATH-MS – spectral libraries generated with 2D LC-MS/MS) |
| 26959854 ^(31)^ | 2016 | Soluble extract | 812 | LC-IMSE |
| 27108548 ^(32)^ | 2016 | Soluble extract (exposure to curcumin) | 36 | 2DE LC-MS/MS  MALDI TOF/TOF |
| 27246823 ^(33)^ | 2016 | Whole membrane  (synthetic cystic fibrosis lung media) | 3613 | LC-MS/MS |
| 27895643 ^(34)^ | 2016 | Secretome  (Azithromycin exposure) | 204 | MudPIT LC-MS/MS |
| 27771372 ^(35)^ | 2017 | Soluble extract  (Ciprofloxacin exposure) | 3251 | LC-MS/MS |
| 28454561 ^(36)^ | 2017 | Soluble extract and secretome  (Grown with glycerol or an equal mixture of C_8_, C_10_, C_12_, C_14_, and C_16_ normal alkanes) | 215^§^ | MudPIT LC-MS/MS |
| 28791256 ^(37)^ | 2017 | Soluble extract  (Grown in 3-day post fertilization zebrafish larvae) | 1159 (injected larvae), 1276 (larvae exposed by immersion) | LC-MS/MS |
| 28824592 ^(38)^ | 2017 | Soluble extract  (Profiles upon adherence to stainless steel, glass, and polystyrene surfaces) | 930 | SDS-PAGE LC-MS/MS |

Note: Care has been taken to identify as many studies as possible to highlight the full extent of PAO1 proteome profiling within the literature. Many of these studies will overlap in the identities of the proteins identified, and most (but not all) do not attempt to quantify nor attempt to characterize the proteins identified to any great depth.

*****: The condition, if applicable (denoted in parenthesis), describes any notable conditions PAO1 was subjected to prior to proteome profiling.

¶: The proteins that were identified by the authors varied in their level of pre-annotation.

§: The reported proteins were only the proteins of interest, or the proteins differentially regulated.

SUPPLEMENTARY REFERENCES:

1. Nouwens AS*, et al.* (2000) Complementing genomics with proteomics: The membrane subproteome of *Pseudomonas aeruginosa* PAO1. *Electrophoresis* 21(17):3797-3809.

2. Malhotra S, Silo-Suh LA, Mathee K, & Ohman DE (2000) Proteome analysis of the effect of mucoid conversion on global protein expression in Pseudomonas aeruginosa strain PAO1 shows induction of the disulfide bond isomerase, dsbA. *Journal of bacteriology* 182(24):6999-7006.

3. Nouwens AS, Willcox MDP, Walsh BJ, & Cordwell SJ (2002) Proteomic comparison of membrane and extracellular proteins from invasive (PAO1) and cytotoxic (6206) strains of *Pseudomonas aeruginosa*. *PROTEOMICS* 2(9):1325-1346.

4. Nouwens AS*, et al.* (2003) Proteome analysis of extracellular proteins regulated by the las and rhl quorum sensing systems in Pseudomonas aeruginosa PAO1. *Microbiology (Reading, England)* 149(Pt 5):1311-1322.

5. Heim S*, et al.* (2003) Proteome reference map of Pseudomonas putida strain KT2440 for genome expression profiling: distinct responses of KT2440 and Pseudomonas aeruginosa strain PAO1 to iron deprivation and a new form of superoxide dismutase. *Environmental microbiology* 5(12):1257-1269.

6. Blonder J*, et al.* (2004) Global analysis of the membrane subproteome of Pseudomonas aeruginosa using liquid chromatography-tandem mass spectrometry. *Journal of proteome research* 3(3):434-444.

7. Kim EJ, Wang W, Deckwer WD, & Zeng AP (2005) Expression of the quorum-sensing regulatory protein LasR is strongly affected by iron and oxygen concentrations in cultures of Pseudomonas aeruginosa irrespective of cell density. *Microbiology (Reading, England)* 151(Pt 4):1127-1138.

8. Nalca Y*, et al.* (2006) Quorum-sensing antagonistic activities of azithromycin in Pseudomonas aeruginosa PAO1: a global approach. *Antimicrob Agents Chemother* 50(5):1680-1688.

9. Diab F*, et al.* (2006) Succinate-mediated catabolite repression control on the production of glycine betaine catabolic enzymes in Pseudomonas aeruginosa PAO1 under low and elevated salinities. *Microbiology (Reading, England)* 152(Pt 5):1395-1406.

10. Patrauchan MA, Sarkisova SA, & Franklin MJ (2007) Strain-specific proteome responses of Pseudomonas aeruginosa to biofilm-associated growth and to calcium. *Microbiology (Reading, England)* 153(Pt 11):3838-3851.

11. Imperi F*, et al.* (2009) Analysis of the periplasmic proteome of *Pseudomonas aeruginosa*, a metabolically versatile opportunistic pathogen. *PROTEOMICS* 9(7):1901-1915.

12. Choi D-S*, et al.* (2011) Proteomic analysis of outer membrane vesicles derived from Pseudomonas aeruginosa. *PROTEOMICS* 11(16):3424-3429.

13. Damron FH*, et al.* (2011) Vanadate and triclosan synergistically induce alginate production by Pseudomonas aeruginosa strain PAO1. *Mol Microbiol* 81(2):554-570.

14. Lecoutere E*, et al.* (2012) A theoretical and experimental proteome map of *Pseudomonas aeruginosa* PAO1. *MicrobiologyOpen* 1(2):169-181.

15. Toyofuku M, Roschitzki B, Riedel K, & Eberl L (2012) Identification of proteins associated with the Pseudomonas aeruginosa biofilm extracellular matrix. *Journal of proteome research* 11(10):4906-4915.

16. Damron FH*, et al.* (2012) Analysis of the Pseudomonas aeruginosa regulon controlled by the sensor kinase KinB and sigma factor RpoN. *Journal of bacteriology* 194(6):1317-1330.

17. Massier S*, et al.* (2012) Adaptation of Pseudomonas aeruginosa to a pulsed light-induced stress. *Journal of applied microbiology* 112(3):502-511.

18. Hare NJ*, et al.* (2012) Proteomic profiling of Pseudomonas aeruginosa AES-1R, PAO1 and PA14 reveals potential virulence determinants associated with a transmissible cystic fibrosis-associated strain. *BMC microbiology* 12:16.

19. Casabona MG, Vandenbrouck Y, Attree I, & Couté Y (2013) Proteomic characterization of *Pseudomonas aeruginosa* PAO1 inner membrane. *PROTEOMICS* 13(16):2419-2423.

20. Cierniak P, Jubner M, Muller S, & Bender K (2013) Insights into mechanisms and proteomic characterisation of Pseudomonas aeruginosa adaptation to a novel antimicrobial substance. *PLoS One* 8(7):e66862.

21. Dagorn A*, et al.* (2013) Gamma-aminobutyric acid acts as a specific virulence regulator in Pseudomonas aeruginosa. *Microbiology (Reading, England)* 159(Pt 2):339-351.

22. Kumari H*, et al.* (2014) LTQ-XL mass spectrometry proteome analysis expands the Pseudomonas aeruginosa AmpR regulon to include cyclic di-GMP phosphodiesterases and phosphoproteins, and identifies novel open reading frames. *Journal of proteomics* 96:328-342.

23. Park AJ, Surette MD, & Khursigara CM (2014) Antimicrobial targets localize to the extracellular vesicle-associated proteome of Pseudomonas aeruginosa grown in a biofilm. *Front Microbiol* 5:464.

24. Sarkisova SA*, et al.* (2014) A Pseudomonas aeruginosa EF-hand protein, EfhP (PA4107), modulates stress responses and virulence at high calcium concentration. *PLoS One* 9(2):e98985.

25. Kwon T, Huse HK, Vogel C, Whiteley M, & Marcotte EM (2014) Protein-to-mRNA ratios are conserved between Pseudomonas aeruginosa strains. *Journal of proteome research* 13(5):2370-2380.

26. Kubacka A*, et al.* (2014) Understanding the antimicrobial mechanism of TiO(2)-based nanocomposite films in a pathogenic bacterium. *Scientific reports* 4:4134.

27. Park AJ*, et al.* (2014) A temporal examination of the planktonic and biofilm proteome of whole cell Pseudomonas aeruginosa PAO1 using quantitative mass spectrometry. *Molecular & cellular proteomics : MCP* 13(4):1095-1105.

28. Herbst FA*, et al.* (2015) Major proteomic changes associated with amyloid-induced biofilm formation in Pseudomonas aeruginosa PAO1. *Journal of proteome research* 14(1):72-81.

29. Couto N, Schooling SR, Dutcher JR, & Barber J (2015) Proteome profiles of outer membrane vesicles and extracellular matrix of Pseudomonas aeruginosa biofilms. *Journal of proteome research* 14(10):4207-4222.

30. Penesyan A*, et al.* (2015) Genetically and Phenotypically Distinct Pseudomonas aeruginosa Cystic Fibrosis Isolates Share a Core Proteomic Signature. *PloS one* 10(10):e0138527.

31. Lassek C, Berger A, Zuhlke D, Wittmann C, & Riedel K (2016) Proteome and carbon flux analysis of Pseudomonas aeruginosa clinical isolates from different infection sites. *Proteomics* 16(9):1381-1385.

32. Sethupathy S*, et al.* (2016) Proteomic analysis reveals modulation of iron homeostasis and oxidative stress response in Pseudomonas aeruginosa PAO1 by curcumin inhibiting quorum sensing regulated virulence factors and biofilm production. *Journal of proteomics* 145:112-126.

33. Kamath KS*, et al.* (2016) Pseudomonas aeruginosa Cell Membrane Protein Expression from Phenotypically Diverse Cystic Fibrosis Isolates Demonstrates Host-Specific Adaptations. *Journal of proteome research* 15(7):2152-2163.

34. Leal T*, et al.* (2016) Azithromycin Attenuates Pseudomonas-Induced Lung Inflammation by Targeting Bacterial Proteins Secreted in the Cultured Medium. *Frontiers in immunology* 7:499.

35. Peng J, Cao J, Ng FM, & Hill J (2017) Pseudomonas aeruginosa develops Ciprofloxacin resistance from low to high level with distinctive proteome changes. *Journal of proteomics* 152:75-87.

36. Grady SL*, et al.* (2017) A comprehensive multi-omics approach uncovers adaptations for growth and survival of Pseudomonas aeruginosa on n-alkanes. *BMC Genomics* 18(1):334.

37. Diaz-Pascual F, Ortiz-Severin J, Varas MA, Allende ML, & Chavez FP (2017) In vivo Host-Pathogen Interaction as Revealed by Global Proteomic Profiling of Zebrafish Larvae. *Front Cell Infect Microbiol* 7:334.

38. Guilbaud M*, et al.* (2017) Proteomic Response of Pseudomonas aeruginosa PAO1 Adhering to Solid Surfaces. *Front Microbiol* 8:1465.

39. Stover CK*, et al.* (2000) Complete genome sequence of *Pseudomonas aeruginosa* PAO1, an opportunistic pathogen. *Nature* 406(6799):959-964.

40. Teitzel GM*, et al.* (2006) Survival and Growth in the Presence of Elevated Copper: Transcriptional Profiling of Copper-Stressed Pseudomonas aeruginosa. *Journal of bacteriology* 188(20):7242-7256.
