# Supplementary material for: Proteome profiling of Pseudomonas aeruginosa PAO1 identifies novel responders to copper stress
Source: BMC Microbiol. 2019 Apr 1;19:69. doi: 10.1186/s12866-019-1441-7 (PMC6444534; doi:10.1186/s12866-019-1441-7)
Supplement: Supplementary file 2 — P. aeruginosa PAO1 gene product distributions. (DOCX 596 kb) [file 12866_2019_1441_MOESM2_ESM.docx]

**Additional file 2**


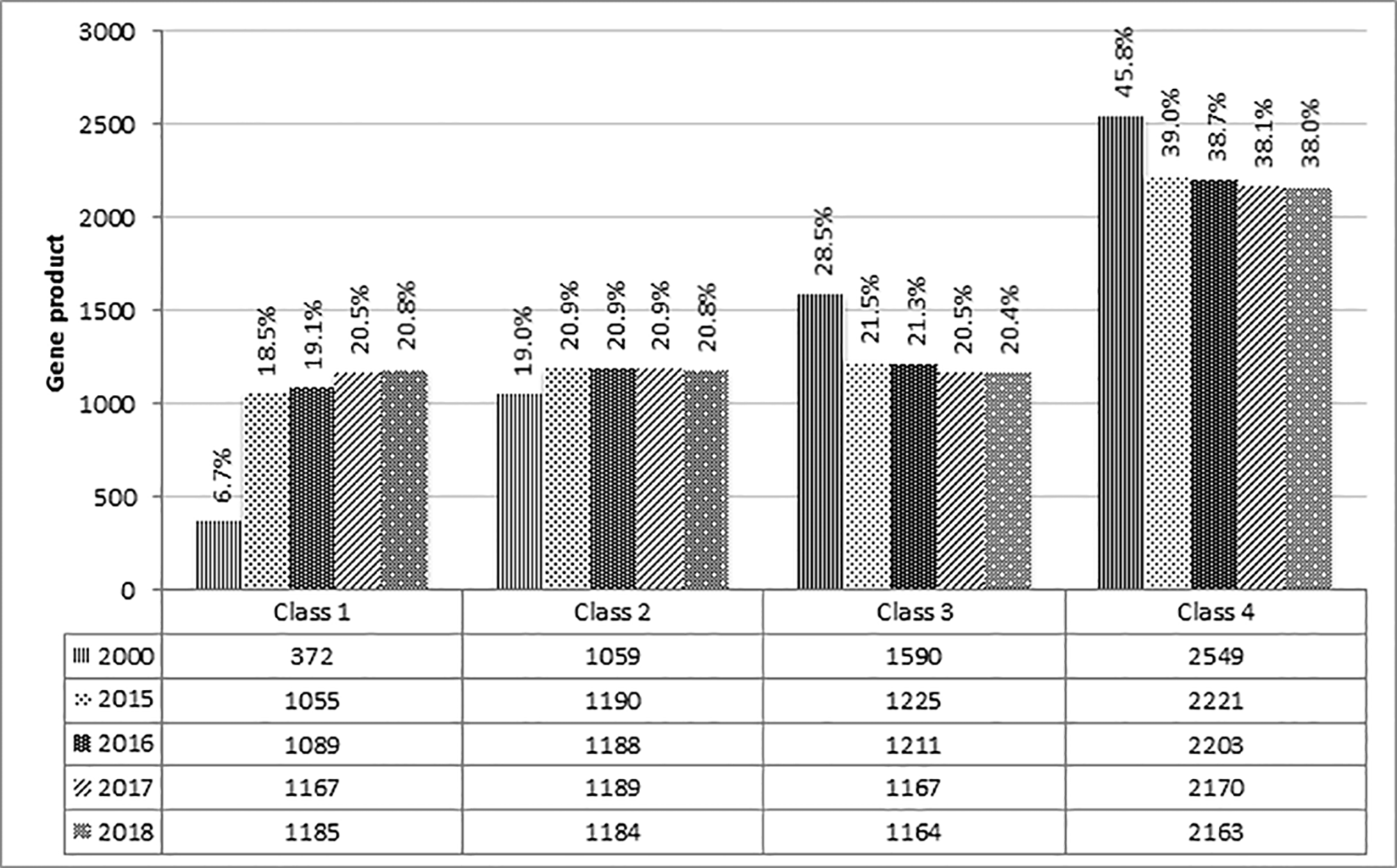


**Gene products distributions across product name confidence ratings in the year 2000 ^(39)^ compared to 2015, 2016, 2017, and 2018 (data extracted from PGD 6^th^ August 2015, 6^th^ September 2016, 19^th^ September 2017, and 30^th^ April 2018, respectively).** Classes are as follows: Class 1 – Function experimentally demonstrated in *P. aeruginosa*, class 2 – Function of a highly similar gene experimentally demonstrated in another organism (and gene context consistent in terms of pathways involved in, if known), class 3 – Function proposed based on presence of conserved amino acid motif, structural feature or limited sequence similarity to an experimentally demonstrated gene, class 4 – Homologs of previously reported genes of unknown function, or no similarity to any previously reported genes. The percentage at the top of each bar refers to the proportion of gene products in that category from that year’s total.
