# Supplementary material for: Proteome profiling of Pseudomonas aeruginosa PAO1 identifies novel responders to copper stress
Source: BMC Microbiol. 2019 Apr 1;19:69. doi: 10.1186/s12866-019-1441-7 (PMC6444534; doi:10.1186/s12866-019-1441-7)
Supplement: Supplementary file 3 — P. aeruginosa growth curve under exposure to CuSO4. (DOCX 17 kb) [file 12866_2019_1441_MOESM3_ESM.docx]

**Additional file 3**

**PAO1 growth in CuSO_4_.** The growth study involved PAO1 grown in M9 medium with or without 50µM CuSO_4_ addition. OD_600_ spectrophotometric readings were taken hourly from hour 4 of growth until hour 21.
