# Supplementary material for: Proteome profiling of Pseudomonas aeruginosa PAO1 identifies novel responders to copper stress
Source: BMC Microbiol. 2019 Apr 1;19:69. doi: 10.1186/s12866-019-1441-7 (PMC6444534; doi:10.1186/s12866-019-1441-7)
Supplement: Supplementary file 5 — Proteins mapped to COG functional categories that are used to represent major biological functions. (DOCX 1388 kb) [file 12866_2019_1441_MOESM5_ESM.docx]

**Additional file 5**

**COG functional categories used to represent major biological functions. This distribution map highlights the COG % distribution for all proteins in the soluble and membrane fractions and their respective unique proteins (unique soluble, and unique membrane), and also contains the % distribution for 5698 proteins of PAO1 (derived from the PGD).** The COG functional categories are: **A** – RNA processing and modification, **B** – chromatin structure and dynamics, **C** – energy production and dynamics, **D** – cell cycle control, cell division, and chromatin partitioning, **E** – amino acid transport and metabolism, **F** – nucleotide transport and metabolism, **G** – carbohydrate transport and metabolism, **H** – coenzyme transport and metabolism, **I** – lipid transport and metabolism, **J** – translation, ribosomal structure, and biogenesis, **K** – transcription, **L** – replication, repair, and recombination, **M** – cell wall/membrane/envelope biogenesis, **N** – cell motility, **O** – post-translational modification, protein turnover, and chaperones, **P** – inorganic ion transport, **Q** – secondary metabolite biosynthesis, transport, and catabolism, **R** – general function prediction only, **S** – function unknown, **T** – signal transduction mechanisms, **U** – intracellular trafficking, secretion, and vesicular transport, **V** – defense mechanisms, **W** – extracellular structures, **X** – mobilome: prophages, and transposons, **Y** – nuclear structure, **Z** – cytoskeleton, and **§** – no COG assignment.
