# Supplementary material for: Proteome profiling of Pseudomonas aeruginosa PAO1 identifies novel responders to copper stress
Source: BMC Microbiol. 2019 Apr 1;19:69. doi: 10.1186/s12866-019-1441-7 (PMC6444534; doi:10.1186/s12866-019-1441-7)
Supplement: Supplementary file 6 — Shared observations of this study (proteomic) and the transciptomic study of Teitzel et al. (DOCX 25 kb) [file 12866_2019_1441_MOESM6_ESM.docx]

**Additional file 6**


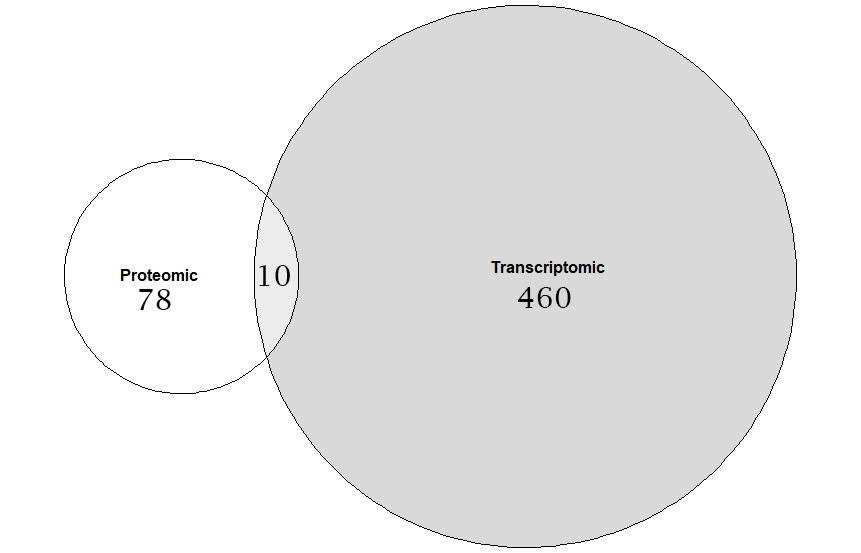


**Shared observations of this study (proteomic) and the study of Teitzel et al ^(40)^ (transcriptomic).** Statistically significant changes in either protein abundance (this study) or transcript abundance ^(40)^ were reported independently. 10 products were observed to be shared between the two studies (Additional file 3).
