# Supplementary material for: Proteome profiling of Pseudomonas aeruginosa PAO1 identifies novel responders to copper stress
Source: BMC Microbiol. 2019 Apr 1;19:69. doi: 10.1186/s12866-019-1441-7 (PMC6444534; doi:10.1186/s12866-019-1441-7)
Supplement: Supplementary file 7 — SWATH-MS variable windows across the 400–1250 m/z range. (DOCX 16 kb) [file 12866_2019_1441_MOESM7_ESM.docx]

**Additional file 7**

**SWATH-MS variable windows (100) across the 400-1250 *m/z* range.**
